# Natural and engineered cyclodipeptides: Biosynthesis, chemical diversity, and engineering strategies for diversification and high-yield bioproduction.

**DOI:** 10.1016/j.engmic.2022.100067

**Published:** 2022-12-24

**Authors:** Wahyu Setia Widodo, Sonja Billerbeck

**Affiliations:** a Department of Molecular Enzymology, Groningen Biomolecular Sciences and Biotechnology Institute, University of Groningen, Groningen, The Netherlands; b Department of Molecular Microbiology, Groningen Biomolecular Sciences and Biotechnology Institute, University of Groningen, Groningen, The Netherlands

**Keywords:** Cyclodipeptides, Non-ribosomal peptide synthetase, Cyclodipeptides synthase, Pulcherriminic acid, Thaxtomin a, Metabolic engineering, Synthetic biology

## Abstract

Cyclodipeptides are diverse chemical scaffolds that show a broad range of bioactivities relevant for medicine, agriculture, chemical catalysis, and material sciences. Cyclodipeptides can be synthesized enzymatically through two unrelated enzyme families, non-ribosomal peptide synthetases (NRPS) and cyclodipeptide synthases (CDPSs). The chemical diversity of cyclodipeptides is derived from the two amino acid **side** chains and the modification of those side-chains by cyclodipeptide tailoring enzymes. While a large spectrum of chemical diversity is already known today, additional chemical space - and as such potential new bioactivities - could be accessed by exploring yet undiscovered NRPS and CDPS gene clusters as well as via engineering. Further, to exploit cyclodipeptides for applications, the low yield of natural biosynthesis needs to be overcome. In this review we summarize current knowledge on NRPS and CDPS-based cyclodipeptide biosynthesis, engineering approaches to further diversity the natural chemical diversity as well as strategies for high-yield production of cyclodipeptides, including a discussion of how advancements in synthetic biology and metabolic engineering can accelerate the translational potential of cyclodipeptides.

## Graphical abstract

**Figure:**
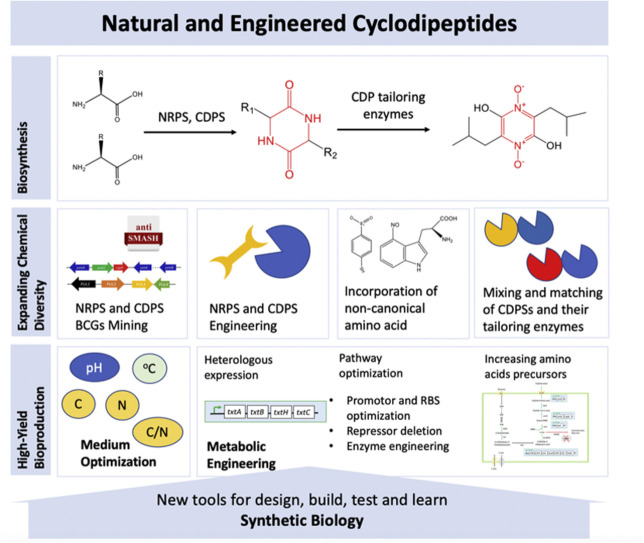

## 1 Introduction

**Fig. 1:**
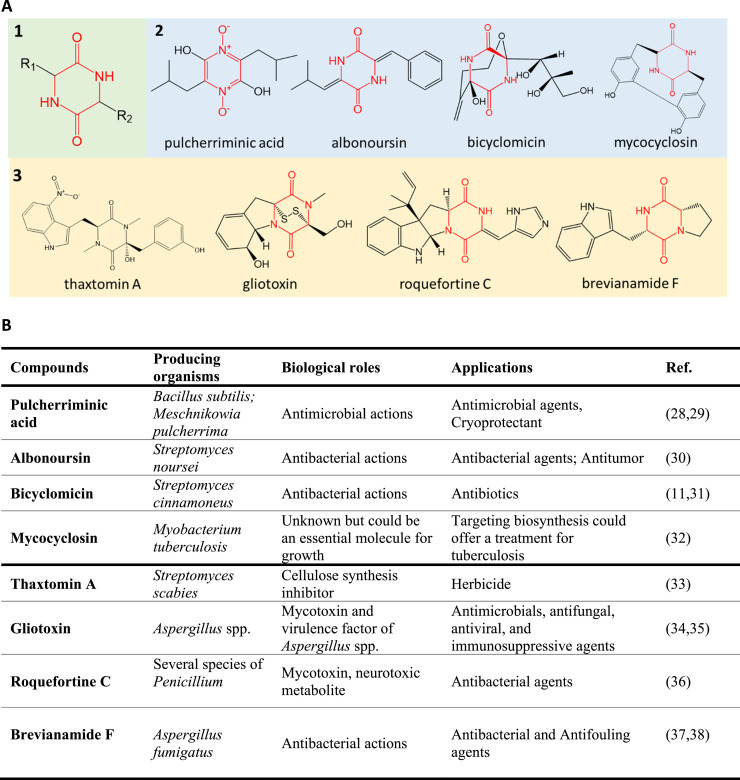
**Structure and functions of several cyclodipeptides and their derivates. A.** 1) general core structure of CDPs; 2) CDPs from CDPS pathway; 3) CDPs from NRPS pathway. **B.** Overview of the producing organisms and the biological role and potential application of the various compounds listed in A. Refs. [11], [28], [29], [30], [31], [32], [33], [34], [35], [36], [37], [38] are cited within this figure.

Cyclodipeptides, also known as cyclic dipeptides or 2,5-diketopiperazines (2,5-DKPs), are the smallest cyclic peptides present in nature, just containing two amino acids. Cyclic peptides have enhanced properties when compared to their linear analogs and are considered promising scaffolds for pharmaceutical applications and material science due to their structural diversity, rigidity, and stability against proteolysis [1,2]. Cyclodipeptides have a wide variety of biological functions, including antibacterial [3], antifungal [4,5], anticancer [6], [7], [8], anti-Alzheimer in vitro [9] and antioxidant properties [10] **(**Fig. 1**)**. Several cyclodipeptides are currently used as pharmaceuticals such as bicyclomycin and plinabulin (under clinical trial) [11,12]. In addition, in synthetic organic chemistry, cyclodipeptide derivatives have been considered organo-catalysts and chiral auxiliaries [1].

Cyclodipeptides can be prepared by standard chemical reactions or produced biosynthetically by organisms. In a living organism, cyclodipeptides are synthesized by two unrelated enzyme families: either by non-ribosomal peptide synthetase (NRPS) or by cyclodipeptide synthase (CDPS) [13], [14], [15]. NRPSs and CDPSs are often associated with a specific biosynthetic gene cluster encoding additional enzymes that are responsible for cyclodipeptide scaffold modifications. This so-called “tailoring” allows nature to access a great chemical and functional diversity **(**Fig. 1**)** [16,17].

Many cyclodipeptides have been characterized for human-centric applications, but the biological function of cyclodipeptides in their producing microorganisms is less well understood. In bacteria, cyclodipeptides are mainly used as signaling molecules in intraspecies communication via quorum sensing [18,19] as well as for interspecies signaling [20]. Moreover, cyclodipeptides constitute a class of interkingdom signals due to their ability to regulate both bacterial and eukaryotic cells [21,22]. In mammals, cyclodipeptides can enter the human brain and operate on glial cells to regulate a behavioral transition between homeostatic and inflammatory states [23]. Regarding this function, since cyclodipeptides are blood-brain barrier permeable, they have been designed to facilitate the passage of physiologically active substances across the blood-brain barrier [24]. The ability of a dipeptide in its cyclized form to enter the blood-brain barrier might be partly explained by its reduced number of hydrogen bonds. For instance, cyclic glycine-glycine is predicted to be 100-fold more permeable to the blood-brain barrier compared to its linear form because the number of hydrogen bond is reduced from 8 to 4 [25,26].

In addition, a recent study indicates that cyclodipeptides act as pheromones and are involved in intra-specific (male-male) communication in terrestrial vertebrates [27].

The significance of cyclodipeptides for biotechnological, agricultural, and medical applications and their interesting natural function make them fascinating subjects to study. Some recent reviews have covered their chemical diversity, and biological functions [13,14,39,40].

Here we review recent advances in understanding the biosynthesis of cyclodipeptides, strategies for expanding their chemical diversity, and strategies that can enhance their production yield through fermentation optimization and metabolic engineering. Our review also includes an outlook on how advancements in the field of synthetic biology could be applied to facilitate and accelerate the valorization of cyclodipeptides.

## 2 Biosynthesis of cyclodipeptides

Cyclodipeptides are synthesized by either non-ribosomal peptide synthetase (NRPS) or cyclodipeptide synthase (CDPS). These two unrelated enzyme families vary in protein size, substrate utilization, and reaction processes [14,41].

### 2.1 Cyclodipeptides produced by NRPS

NRPS are modular multidomain enzymes of more than 100 kDa size that catalyze the synthesis of peptides found mainly in fungi and bacteria [41], and in rare cases in Archaea [42]. Only a fraction of NRPSs catalyze the formation of the herein discussed cyclodipeptides. Most of NRPSs catalyze the biosynthesis of longer peptides such as cyclosporine, daptomycin or vancomycin, and others.

NRPS genes are often located within a gene cluster, which usually consists of a core NRPS, several tailoring enzymes, a transcriptional regulator, and a transporter [18,43]. For example, the gene cluster encoding for gliotoxin production in *Aspergillus* species – a well-studied cyclodipeptide that is involved in the virulence of *A. fumigatus* – encodes for the core NRPS enzyme (*gliP*), a transcription factor (*gliZ*), a transporter (*gliA*), and several tailoring enzymes of known and unknown function (*gliGFKNTC*) [43,44].

**Fig. 2:**
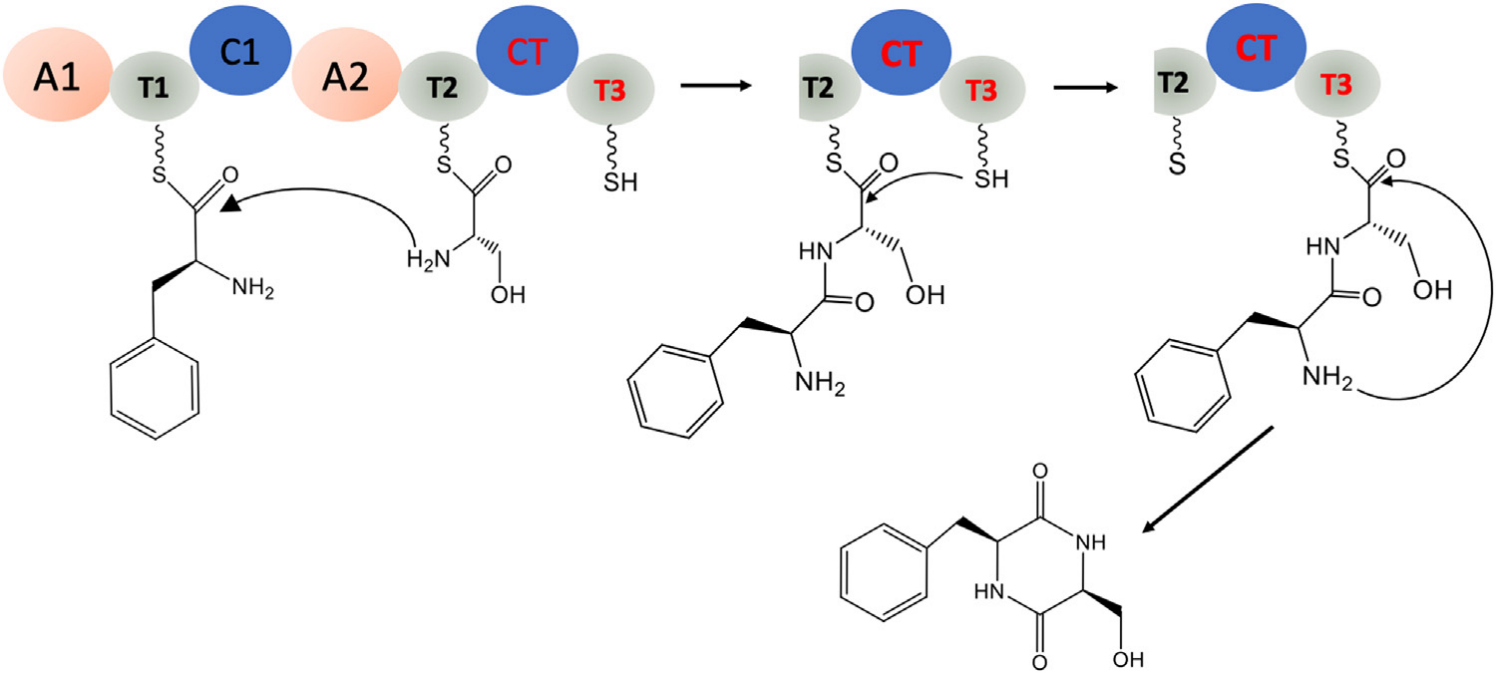
Cyclodipeptide synthesis by the NRPS GliP: the synthesis of cyclo(L-Phe-l-Ser), an intermediate chemical in gliotoxin biosynthesis. GliP consists of two modules ([1] and [2]), and in total two adenylation domains (A), two condensation domains (C), and three thiolation (T) domains. Note: The cyclization step in GliP biosynthesis is catalyzed by two terminal domains: the condensation like domain (CT) and an additional thiolation domain (T3). The last T domain is not always present in NRPSs (see also Fig. 3) [45] but can be found in several putative NRPS gene clusters in fungal genomes [43,46]. The figure is adapted and reproduced from [43].

The core NRPS enzymes utilize free amino acids as their substrates and this differs from the later discussed CDPSs that use aminoacyl-tRNA **(**Fig. 2). The module and domain architecture of NRPSs is well understood and has been excellently summarized here [41]. In brief, a typical NRPS is composed of various modules, each being responsible for the incorporation of a single amino acid into the growing peptide chain. Each module has three essential domains: the adenylation (A) domain, the peptidyl carrier protein (PCP) domain (also known as the thiolation (T) domain), and the condensation (C) domain [47]. The amino acid monomer is selected, activated, and loaded onto the T domain by the A domain **(**Fig. 2). The thiol group of the T domain's 4′-phosphopantetheine arm (ppant) acts as nucleophile and attacks the carboxyl group of the adenylated amino acid resulting in the formation of aminoacylthioester [48,49]. The C domain catalyzes the subsequent formation of peptide bonds between two neighboring T-bound aminoacyl intermediates. The cyclization step in GliP biosynthesis is catalyzed by two terminal domains: the condensation like domain (CT) and additional thiolation domain (T3) **(**Fig. 2**)**. Of note, even though Guo *et al*. (2012) reported that the macrocyclization of linear peptides in a range of fungal multi-module NRPS is catalyzed by a CT domain without an additional T domain [45], the conserved terminal domain in GliP (CT-T3) is also found in other putative NRPS gene clusters in fungal genomes [43,46]. In Bacteria, the terminal module of an NRPS assembly lines typically contains a thioesterase (TE) domain to function as release catalyst and frees the NRPS enzyme for further synthesis. This domain catalyzes either hydrolysis or, more typically, cyclization of the peptide to catalyze release from the peptide from NRPS [48].

Cyclodipeptide scaffolds generated by NRPSs are further chemically diversified by tailoring enzymes resulting in the final cyclodipeptide metabolites [50]. Many cyclodipeptides and their derivates, including thaxtomin A [51], gliotoxin [52], brevianamide F [37], sirodesmin PL [53], erythrochelin [54], roquefortine C [36], ergotamine [55], and acetylaszonalenin [56] are produced through their own dedicated NRPS pathways.

**Fig. 3:**
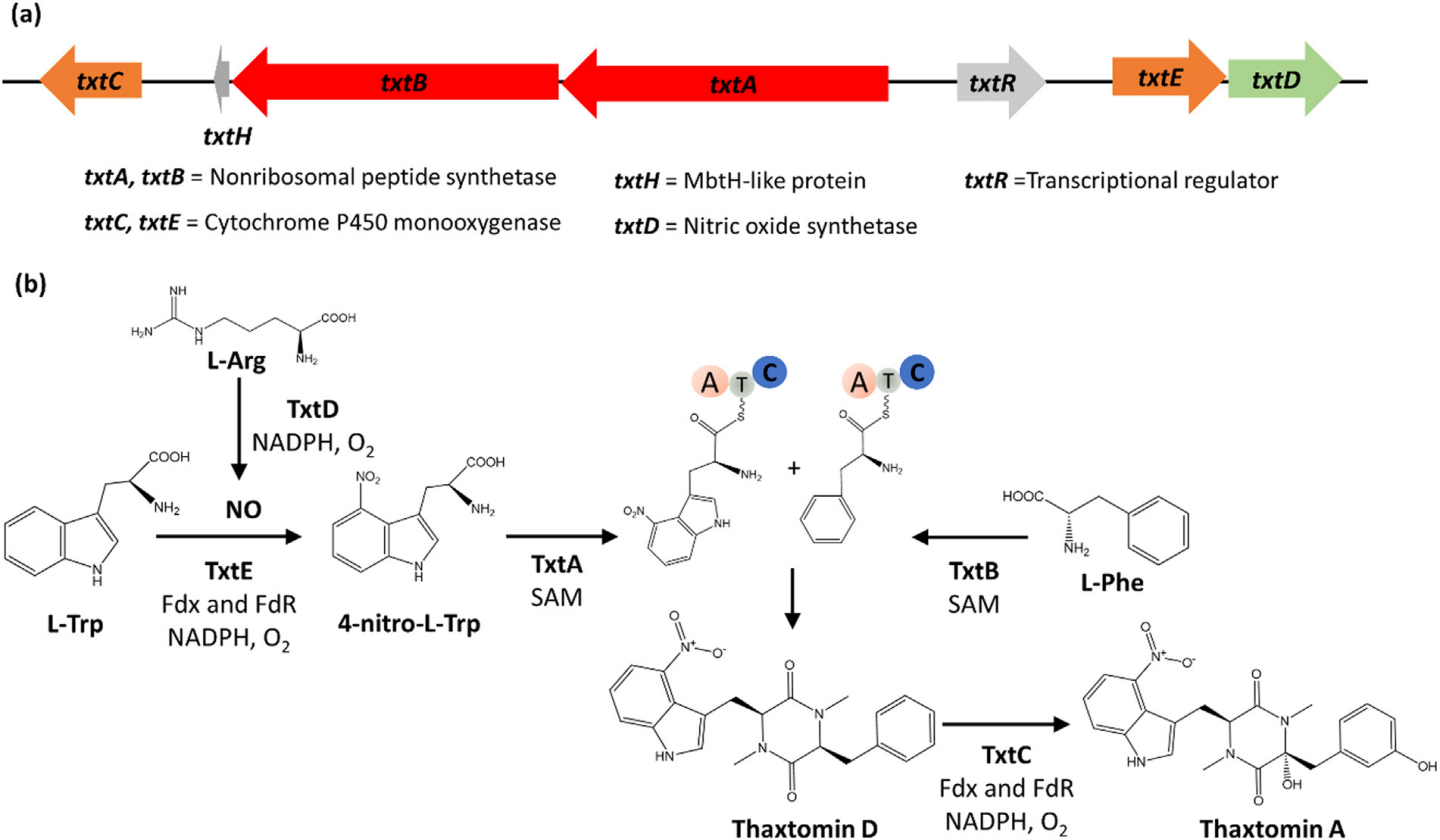
**Thaxtomin A biosynthesis. A.** The gene cluster for thaxtomin A biosynthesis: txtABCDE, txtR (regulatory protein), and txtH (communication protein). **B.** The biosynthesis of thaxtomin A starts with l-arg, l-phe, and l-trp as the precursors. The figure was reproduced from [68].

To give an overview of gene clusters and formation of a final cyclodipeptide molecule by an NRPS-based biosynthetic pathway, we use thaxtomin A biosynthesis as an example **(**Fig. 3). Thaxtomin A is one of the best-studied cyclodipeptides that is produced via the NRPS pathway. Thaxtomin A and its derivatives (in their sum called thaxtomins) are virulence factors produced by many plant-pathogenic *Streptomyces* strains. Their potent natural herbicidal activity – which is based on their inhibition of cellulose biosynthesis – and inherent biodegradability make them key targets for bioherbicide development [57,58].

Thaxtomin A is a cyclo(n-nitro-l-tryptophan-l-phenylalanine), a cyclodipeptide that incorporates the non-canonical amino acid (ncAA) n-nitro-l-tryptophan. Thaxtomin A and its related analogs are synthesized by a highly conserved 18.3 kb gene cluster in *Streptomyces spp* comprising seven genes: *txtA, txtB, txtC, txtD, txtE, txtH,* and *txtR* (Fig. 3)*.* Biosynthesis relies on the three precursor amino acids l-arginine (L-arg), l-tryptophan (L-trp), and l-phenylalanine (L-phe) and is initiated by the oxidation of l-arg to form nitric oxide (NO) and citrulline catalyzed by the NO synthase (TxtD) [59]. The NO is then utilized by the cytochrome P450 monooxygenase TxtE to catalyze the 4-nitration of l-trp, resulting in l-4-nitrotryptophan in the presence of O_2_, NADPH, and electron transfer components [60]. l-phe and the l-4-nitrotryptophan are then assembled to produce the first cyclodipeptide backbone (thaxtomin D) catalyzed by the two NRPSs, TxtA and TxtB [61,62]. Thaxtomin A is then formed after consecutive hydroxylation of the l-phe-carbon and the phenyl group in the cyclodipeptide scaffold by the cytochrome P450 monooxygenase TxtC [63], [64], [65]. *TxtR* encodes the cluster-situated regulator (CSR) and belongs to the AraC/XylS family of transcriptional regulators (AFTRs) [66,67]. Furthermore, txtH is a gene that encodes an MbtH-like protein, which promotes NRPS solubility and adenylation domain activity of TxtA and TxtB. [68].

Besides being synthesized by specialized NRPS-based biosynthetic pathways, in a few cases, cyclodipeptides are synthesized by the premature release of dipeptidyl intermediates during the chain elongation process of a longer non-ribosomal peptide. For example, *in Bacillus brevis*, cyclo(D-Phe-l-Pro) was discovered as an off-pathway product during gramicidin S and tyrocidine A production [69]. Further, during the synthesis of the heptapeptide cyclomarin A by the NRPS CymA in *Siniora arenicola*, also the two cyclodipeptides cyclomarazine A and B are produced. Based on Schultz et al.*,* it is still unclear whether the production of these truncated peptides is either generated by the C-terminal thioesterase (TE) CymQ which performs editing function to hydrolyze the truncated dipeptide or by a non-enzymatic mechanism due to ineffective further processing by module 3 of the NRPS CymA [70]. The spontaneous cyclization of dipeptides could be possible if the dipeptides contain amide groups in one terminus and an ester at the other [1].

### 2.2 Cyclodipeptides produced by cyclic dipeptide synthase (CDPSs)

Cyclodipeptides can also be synthesized by an enzyme family called cyclic dipeptide synthase (CDPSs, ∼30 kDa), which has been discovered much later than NRPS [71]. Instead of free amino acids like NRPSs, CDPSs utilize aminoacyl tRNA as their substrates (amino acids loaded onto their respective tRNA). CDPSs catalyze the formation of two peptide bonds between the aminoacyl parts to generate the core cyclodipeptide structure [15,40,71,72]. Similar to the NRPS pathway, the CDPS are arranged in a gene cluster typically consisting of genes encoding for the CDPS, a transporter, cyclodipeptide tailoring enzymes, and a regulator [14,40]. So far, around 12 biosynthetic CDPS-based gene clusters have been experimentally studied [73], [74], [75], [76], [77], [78], [79], [80], [81], [82], [83], [84] and their biocatalytic details have been summarized in a recent review [38].

These gene clusters vary in size from less than 2 kB to about 10 kB, making them among the smallest natural product gene clusters reported so far [40]. The first CDPS-based gene cluster to be identified and characterized was the albonoursin gene cluster from *Streptomyces noursei* in 2002 [85]. The gene cluster consists of just three genes, *albA, albB*, and *albC. AlbC* encodes for the CDPS AlbC that produces cyclo(L-Phe-l-Leu) (short cFL), whereas AlbA and AlbC (the protein products of *albA* and *albC*) are tailoring enzymes that oxidize cFL to yield albonoursin [78,40].

CDPSs have been mainly identified in three bacterial phyla: Actinobacteria, Firmicutes, and Proteobacteria [86]. But, twelve CDPSs have been identified in four eukaryotic phyla: Ascomycota, Annelida, Ciliophora, and Cnidaria, and one archaeon (*Haloterrigena hispanica*) [15,[87], [88], [89]]. The number of putative CDPS discovered in genomic databases has accelerated in recent years, reaching almost 800, and about 400 of them originated from Actinomycetes [14,89,90]. About 100 CDPSs have been experimentally characterized in terms of substrate specificity and product formation [17,71,74,86,87,[90], [91], [92], [93], [94]].

Phylogenetic and structural studies suggest that the CDPS enzyme family can be divided into two distinct subfamilies, called “NYH” and “XYP” subfamilies. The names are derived from sequence preferences in a triad of amino acids within the active site (Residues X40, Y202, and X203 within AlbC numbering) [93]. Besides the sequence preference in this amino acid triad, XYP and NYH enzymes differ structurally in the first half of their Rossman fold. But regardless of the subfamily, the position of the catalytic residues in both families is similar [95,96].

**Fig. 4:**
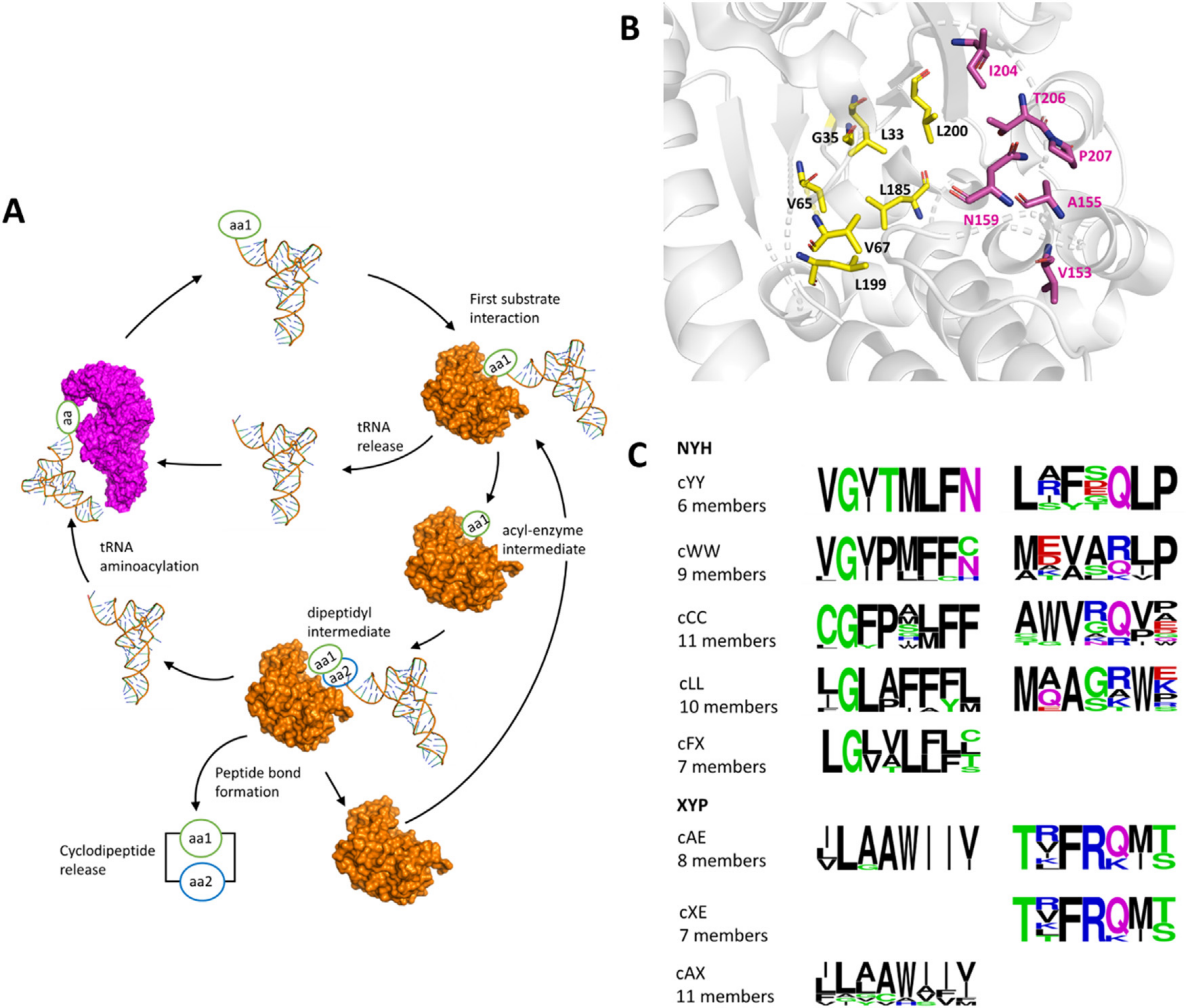
**Overview of the catalytic mechanism of cyclodipeptide synthase. A.** Ping-pong reaction in cyclodipeptide synthesis, CDPSs use two amino-acylated tRNAs and undergo a ‘ping-pong’ reaction to generate a cyclodipeptide**.** The figure was adapted and reproduced from [100]**. B.** Representation of the binding pockets P1 and P2 of AlbC (4Q24). The 8 residues constituting the P1 binding pocket are shown in yellow, and the 7 residues constituting the P2 binding pocket are shown in magenta. **C.** Consensus motifs of aa-tRNA binding pockets P1 and P2 and their specificity towards the first and the second amino acids within the synthesized cyclodipeptide (Note: cYY stands for cyclo(L-Tyr-l-Tyr). These sequence motifs can be used to predict the substrate specificity of each pocket and therefore the final product of the CDPS-catalyzed reaction [99]. P1 and P2 sequences that were used to generate the consensus motifs were retrieved from [86,93].

Since the elucidation of several CDPS crystal structures, the mechanism of action of the enzyme has been well understood (Fig. 4**A**) [96], [97], [98]. All known CDPSs have two surface-accessible binding pockets containing essential residues for substrate recognition and catalysis. The aminoacyl binding sites for the two aa-tRNA substrates are called binding pockets 1 (P1) and 2 (P2). Both pockets are lined with 8 and 7, respectively, residues that determine binding specificity **(**Figs. 4**B and C).**

The sequence motif within both pockets can be used to predict - based on primary structure alone - which substrate (which aa-tRNA) is likely accepted as the first and second amino acid and thus, how the product of a CDPS looks like [93,99] **(**Fig. 4**C)**.

In the first step of catalysis, the aminoacyl group of the first aa-tRNA is transferred to P1’s conserved serine residue upon specific recognition: the tRNA moiety thereby interacts with basic residues in the α-helix 4 to form an aminoacyl–enzyme intermediate. The second aa-tRNA is then bound to the enzyme in P2 [97]. The aminoacyl–enzyme intermediate then reacts with the second aa-tRNA to form a dipeptidyl–enzyme intermediate, which undergoes intramolecular cyclization facilitated by a conserved tyrosine, yielding the CDP scaffold as the final product [98].

The resulting cyclodipeptide scaffolds are then further modified by cyclodipeptide tailoring enzymes encoded in the same gene cluster. These tailoring enzymes include cytochrome P450s, α-ketoglutarate/Fe2+-dependent dioxygenases (α-KGDs), flavin-containing oxidoreductases, α/β-hydrolases, prenyltransferases (PTs) and methyltransferases (MTs), cyclases, and ligases [101,40].

Some of these tailoring enzymes feature unique enzymatic reaction mechanisms – such as challenging intramolecular C—C bond formation [79,80,81] or intermolecular C—N bond formations to achieve functionalization with nucleobases [73] – that could be further developed into biosynthetic tools for non-natural product formation [81,40].

**Fig. 5:**
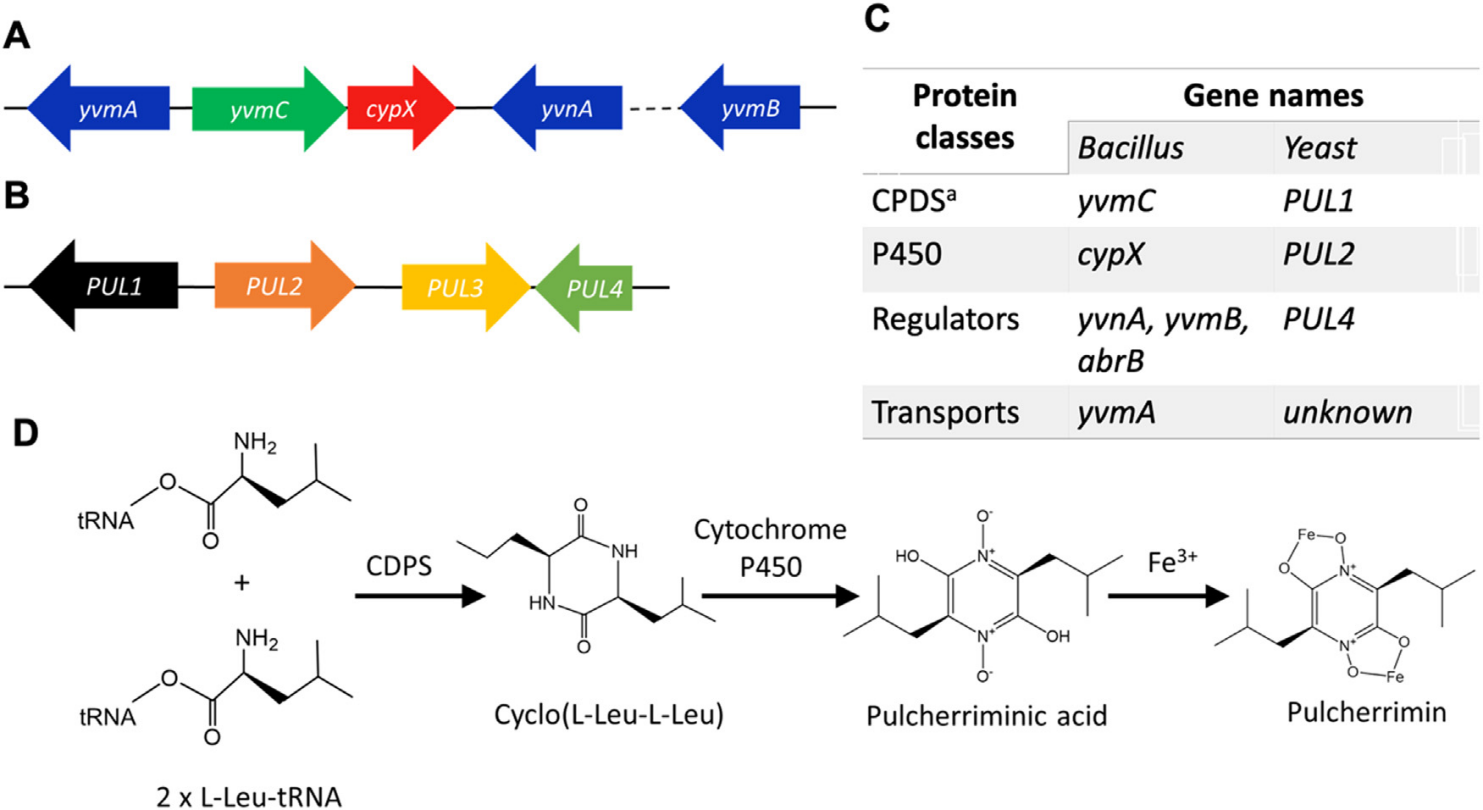
**The biosynthetic gene clusters for pulcherriminic acid production in bacteria and yeast. A.** Biosynthesis in *B. subtilis* is encoded by the two-gene operon *yvmC* and *cypX*, further there are regulators and a transporter. **B.** In *Kluveromyces lactis* and other yeasts, pulcherriminic acid production is encoded by the Pul gene cluster. The Pul gene cluster is phylogenetically distinct from the *Bacillus* gene cluster but seems to contain proteins with similar functions*.***C.** The protein classes encoded by each gene in the cluster. ^a^Note, that the proteins YvmC and Pul1 do not share homology and it needs to be experimentally verified which reaction mechanism Pul1 follows and if it is similar to the CDPS-based reaction [105]. **D.** In *Bacillus*, biosynthesis of pulcherriminic acid is catalyzed by two enzymes (a CDPS and a cytochrome P450 oxygenase) from two leucyl-tRNAs.

One well-studied example of a cyclodipeptide synthesized through the CDPS pathway is pulcherriminic acid **(**Fig. 5**)**. Pulcherriminic acid is an oxidized cyclo(L-Leu–L-Leu, cLL) [102] and is produced by bacteria, mainly *Bacillus* species, or by yeast, particularly by *Metschnikowia* species [[103], [104], [105],^4^]. Pulcherriminic acid is an iron-chelating molecule that can sequester essential iron from the environment [28,29] and as such possesses promising biocontrol activity towards several plants and human pathogens [106,28].

The biosynthesis of pulcherriminic acid has been mainly studied in Bacillus species [106]. The pulcherriminic acid gene cluster comprises five genes: yvmA, yvmB, yvmC, cypX, yvnA **(**Fig. 5**)** [29]. The CDPS YvmC generates cLL using two molecules of leucyl-tRNA [107]. Then, cLL is oxidized by a cytochrome P450 encoded by cypX to generate pulcherriminic acid [108]. Finally, pulcherriminic acid is secreted from the cell through the major facilitator superfamily (MFS) transporter encoded by yvmA. The main transcription factor regulating yvmC-cypX expression is theYvmB, MarR-like regulator encoded by yvmB gene. The function of yvnA is still unknown [109].

Although the discovery of pulcherriminic acid was first described in yeast [110], the corresponding gene cluster encoding for its biosynthesis was only recently identified and characterized in yeast (Pul gene cluster with genes *PUL1, PUL2, PUL3, PUL4,* Fig. 5**B**). [105] Interestingly, no homologous gene clusters could be identified in yeast genomes when using the *Bacillus* gene cluster as a search query. The yeast gene cluster was rather identified by searching for conserved domains from cytochromes P450 (enzymes that could perform the oxidation done by *cypX* in *Bacillus*). This yielded the gene *PUL2* as a hit in *Kluveromyces lactis* (a pulcherrimin pigment producer). Gene deletion and complementation studies revealed *PUL2* and the upstream encoded gene *PUL1* to be essential for pulcherriminic acid production. *PUL1* seems responsible for the formation of cLL, and *PUL2* seems to oxidize cLL into pulcherriminic acid [105]. The other two genes in the operon seem to encode for a regulator and a transporter (PUL4 and PUL3 respectively), based on gene deletion and cross-feeding studies. [105] the *PUL* gene cluster was also identified in the genomes of other pulcherriminic acid-producing yeast [105].

## 3 Expanding the diversity of enzymatically synthesized cyclodipeptides

The so-far known cyclodipeptides point to the huge application potential of this class of molecules. To further this potential, several strategies have been developed to expand the chemical diversity that is currently available to us: This includes the (i) genome-mining and biochemical characterization of novel, natural cyclodipeptide biosynthetic gene clusters (NRPS- and CDPS-based), (ii) engineering of the biosynthetic enzymes, (iii) incorporation non-natural precursors such as non-proteinogenic amino acids, and (iv) combining enzymes from different gene-clusters into new biosynthetic pathways.

### 3.1 Accessing the natural diversity: exploration of novel cyclodipeptide-producing gene clusters and their biochemical characterization

Since the biosynthetic pathways involved in cyclodipeptide production are mainly arranged in gene clusters, genome mining in combination with comparative and functional genomics has been widely applied to explore novel enzymes involved in cyclodipeptide production, as tailoring enzymes can be found in the vicinity of NRPSs and CDPSs [111,112,99]. Most of the currently known chemical diversity can be attributed to the ability to effectively scan microbial genome information.

#### 3.1.1 Genome mining and characterization of NRPSs

NRPS-based gene clusters are significant gene clusters for secondary metabolites production; not only for the production of cyclodipeptides but also for the production of larger non-ribosomal peptides of translational significance [111,113]. As such several tools have been developed for NRPS-based gene cluster mining, including the prediction of the chemistries of the final products: These tools include antiSMASH [114], NP.searcher (for NRPS annotation) [115], and ClustScan [116] and several recent reviews provide an excellent overview on the current status of NRPS-based genome mining and product prediction [[111], [112], [113],^117^].

The gene clusters involved in cyclodipeptide biosynthesis that have been discovered through genome mining include gliotoxin and thaxtomin A [44,62]. Recently, genome mining in *Scedosporium apiospermum* revealed two new NRPS-based gene clusters involved in cyclodipeptide production and tailoring [118]. Genome mining has exponentially expanded our knowledge of sequenced and computationally annotated NRPS-based gene clusters. The actual experimental characterization of the enzymes and the final products as well as their high-yield production has become a new bottleneck in the field. Challenges and advances in characterizing NRPS-based gene clusters have been reviewed here [119,120,121]. Advances toward high-yield production will be addressed in Section 3 of this article.

#### 3.1.2 Genome mining and characterization of CDPS

Also for CDPSs, genome mining is the method of choice to discover new CDPS-encoding genes and tailoring chemistry. The most recent gene clusters that were genome-mined and characterized include streptoazines A to C [84], purincyclamide [75] and cyctetryptomycin A and B [76].

Skinnider et al. built a pipeline to globally search microbial genomes for CDPS-based gene clusters [99]. This includes the prediction of the CDPS substrate specificity based on their P1 and P2 sequence motifs **(**Fig. 4**C)** [93] as well as predicting the final cyclodipeptide chemical structure based on predicted tailoring functions encoded within the gene cluster. Together with Gondry et al.’s effort to characterize a large set of new CDPS substrate specificities [86], this led to the conclusion that out of the 20 possible proteogenic amino acids, only lysine and aspartate have not yet been found to be incorporated by currently characterized CDPS.

Most importantly, CDPS-based genome mining efforts showed that the chemical space of naturally encoded cyclodipeptides is yet experimentally underexplored with many interesting and potentially unique tailoring enzymes to be discovered [99,40].

### 3.2 Expanding the natural diversity by engineering the biosynthesis enzymes

Protein engineering – either rational or by directed evolution – is a powerful tool to expand the catalytic capacities and physicochemical properties of enzymes towards user-defined applications. [122] As such it is also an important means to increase structural diversity within cyclic dipeptides. Additionally, the engineering of individual NRPS and CDPS enzymes contributed to a deeper fundamental understanding of substrate recognition and catalysis.

While the field of NRPS engineering covers three decades – with the first successful engineering. reported in 1995 [123], the field of CDPS engineering is still in its infancies, with a first successful directed evolution experiment reported just recently [124].

#### 3.2.1 Engineering of NRPS

NRPS engineering can be accomplished at several levels of the assembly line's hierarchy: at the levels of modules, domains, and amino acids [41]. The apparent modularity of the NRPS assembly line **(**Fig. 2**)** suggested to protein engineers the possibility to reprogram domains and modules to build novel peptide structures. Stachelhaus et al. already showed in 1995 that it was possible to harness the modularity of NRPS by swapping the A-T didomains in the surfactin NRPS to obtain non-natural peptides [123]. Following this success, many comparable approaches that relied on multiple domain exchanges were published but often the engineering resulted in a decrease in peptide titers, and the non-natural diversity that could be achieved remained limited [41,125]. Recently, Bozhüyük et al. redefined the classical NRPS module boundaries and showed successful modular engineering of NRPSs from Gram-positive and Gram-negative bacteria, that produced both known and artificial non-ribosomal peptides with only moderately reduced titers [126]. The authors proposed defined “exchange units” (XU) that rely on a conserved motif in the C-A linker as a recombination site [126].

Shortly after, a new potential fusion point was identified within the C domain itself that allowed to keep the specificities of the C domain, [127] which had shown to be important for engineering success. [128]

Besides focusing on module and domain exchanges, much attention has been paid to engineering the substrate specificity of the adenylation domains (A domain) of NRPS modules by mutational engineering – this is as the A domain is the gatekeeper for which amino acid is incorporated into the growing peptide [41,125]. This engineering was facilitated by the identification of the so-called “specificity-conferring code” of adenylation domains that allowed to predict which amino acid would be activated by a given A domain [129,130]. Two excellent reviews highlight the numerous engineering efforts performed on NRPS assembly lines [41,125]. Here we focus on those efforts that yielded cyclodipeptides:

Kries et al. engineered an NRPS on the subdomain level and successfully created a chimeric enzyme able to produce programmed cyclodipeptides by swapping short subdomains to transfer specificity from one NRPS module to another [131]. In a complementary approach, the same group used mutational engineering of GrsA to change the substrate specificity of the adenylation domain of GrsA [132]. In both studies [131,132], the authors thereby used just the first two modules (GrsA and GrsB) of the full NRPS assembly line for gramicidin S biosynthesis (a cyclodecapeptide), showing that NRPS-based assembly lines can be disassembled into shorter biosynthesis pathways yielding just cyclodipeptides [131]. The approach of taking individual NRPS modules out of context for catalysis is known as combinatorial biosynthesis and numerous examples of this approach in producing modified non-ribosomal peptides (longer than cyclic dipeptides) have been summarized by Süssmuth et al. [41].

Another exciting example is the switching of communication-mediating (COM) domains (also known as docking domains) to create dipeptide combinations. COM domains are found at the C- and N-termini of partner NRPSs and are involved in protein-protein interactions [133]. These COM domains can also create crosstalk between various biosynthetic systems, resulting in a broader range of combinations [134]. By harnessing this approach, heterologous production and secretion of a designed dipeptide in *S. cerevisiae* as a host organism was shown possible [135].

#### 3.2.2 Engineering of CDPS

There are still few, yet promising studies that show that the engineering of CDPS towards new substrate specificities is possible. Several mutational studies have focused on understanding the substrate specificity of the P1 and P2 pocket, thereby yielding information on the capacity for engineering a CDPS's product formation specificity. For example, Brockmeyer et al. showed that a mutation in P1 of a CDPS from *Nocardiopsis prasine* increased the production of the primary peptide cyclo(L-tyr-l-tyr or cYY) by 10-fold and the production of a secondary product cyclo(L-tyr-l-phe or cYF) by 8-fold when tested via heterologous expression in *E. coli* [90]. Yao et al. showed by systematic mutagenesis within the P1 and P2 pockets of the CDPS DmtB1 from *Streptomyces* spec that the enzymes’ substrate and consequently the product range can be shifted [83]. For example, one residue exchange in P2 of DmtB1 (K155A) increased the production of the primary peptide cWV as well as the production of one of the secondary peptides (cWL). Another residue exchange in P2 (V205M) resulted in reduced cWV production but enhanced cWL production, turning cWL into the primarily produced peptide of this DmtB1 variant [83].

Finally, Sutherland et al. showed the first successful directed evolution of a CDPS towards a new substrate specificity and consequently a new product formation range (from cHP to CFP and cLP) [124]. The authors first solved the crystal structure of Parcu-CDPS from *Parcubacteria bacterium* RAAC4_OD1_1, one of the only two known CDPSs accepting histidine as substrate, and used two rounds of structure-guided directed evolution with a single-residue and double-residue mutational library to reach their goal [124].

### 3.3 Expanding the natural diversity by incorporation of unnatural precursor analogs such as non-proteinogenic amino acids

The substrate promiscuity of natural and engineered NRPSs and CDPSs has made the incorporation of non-natural precursors such as non-proteinogenic amino acids a viable option to increase the chemical diversity of cyclodipeptides. The growing availability of genetic engineering tools to create (heterologous) producer strains (e.g. *E. coli*) deficient for the production of natural substrates such as amino acids has enhanced studies in this field over the last decade [41]. Robust growth of these strains can be achieved by co-feeding the amino acid the strain is deficient for, while during the cyclodipeptide production phase the amino acid can be replaced by a user-defined non-proteinogenic one to force the strain to incorporate it into the final product.

#### 3.3.1 Non-natural substrate usage in NRPS

The use of non-natural substrates in combination with NRPS-based machinery has a long tradition, but these efforts have mainly focused on the diversification of longer non-ribosomal peptides such as cyclosporine, daptomycin, vancomycin, and others. These efforts have been summarized in detail by Süssmuth et al. [41]. Here we only highlight those studies that use non-natural substrates for the diversification of NRPS-produced cyclodipeptides.

Kries et al. engineered the adenylation domain of GrsA, a phenylalanine-specific A domain (PheA) derived from the core NRPSs of gramicidin S biosynthesis, via saturation mutagenesis of eight active site residues to efficiently activate non-natural aromatic amino acids functionalized with azide and alkyne groups such as O-propargyl-l-tyrosine [136]. Azides and alkynes readily undergo bio-orthogonal click reactions and provide a powerful means for labeling, and modifying biologically active peptides [137]. Interestingly, a single residue exchange was sufficient to “switch” the enzyme's specificity from the original substrate phenylalanine to the new substrate O-propargyl-l-tyrosine. The authors then combined the engineered GrsA with a second module of the gramicidin NRPS GrsB1 (activating proline) to create a dipeptide synthase able to catalyze the formation. Besides feeding non-natural substrates into the NRPS enzyme, non-natural substrates can also be channeled into the biosynthetic pathway at the level of the tailoring enzymes. For example, Ouchaou et al. produced roquefortine D analogs in engineered strains of *Penicillium chrysogenum* [138]. They externally supplied a variety of chemically synthesized cyclic dipeptides analogs – so-called calledhistidyltryptophanyldiketopiperazine (HTD) analogs, cyclic dipeptides formed from histidine and tryptophan by the core NRPS of roquefortine biosynthesis RoqA – to a strain lacking the RoqA (ΔroqA) resulting in the production of a variety of novel roquefortine D derivatives depending on the nature of the synthesized HTD supplied.

Besides these engineered systems, there are also reports of NRPSs that naturally incorporate non-proteinogenic amino acids as part of their biosynthetic pathways. For example, 4-nitrotryptophan is used as a substrate of txtA in thaxtomin A biosynthesis [61], and anthranilic acid is a substrate of AnaPS in acetylaszonalenin biosynthesis [56].

#### 3.3.2 Non-natural substrate usage in CDPSs

Canu et al. and Sutherland et al. recently showed that several CDPSs are sufficiently promiscuous in substrate usage to accept tRNAs loaded with non-canonical amino acids (ncAA), indicating that this approach may be utilized to synthesize a wide range of new-to-nature molecules [139,124,14].

Canu et al. used an in vivo set-up in *E. coli* to express their CDPS enzymes and load the ncAA onto the tRNAs. They used various CDPS enzymes with different substrate specificities and analogs of proline, tyrosine, tryptophan, and some aliphatic amino acids. The analogs included various chemical modifications, ranging from simple substitutions like fluorination, hydroxylation, and methylation to more complicated modifications such addition of “clickable” azido groups. From the 60 ncAAs used, 26 ncAAs have been incorporated, resulting in the creation of about 200 ncAA-containing cyclodipeptides. The majority of those CDPs had never been synthesized previously [139].

Sutherland et al. showed in an in vitro reaction using *E. coli*-derived tRNA pools, [140] that the two Histidine-accepting CDPS Para-CDPS (from *Parabacteroides* sp. 20_3) and Parcu-CDPS (from *Parcubacteria bacterium* RAAC4_OD1_1) would incorporate various histidine analogs such as H-β-(2-Thiazolyl)-alanine, 3-(2-pyridyl)-l-alanine and β-(1,2,4-Triazol-3-yl)-DL-alanine [124]. These studies also showed that various histidine analogs could not be incorporated [124].

One challenge in incorporating ncAA into CDPS-produced cyclodipeptide scaffolds is the requirement for non-canonical aminoacyl tRNAs as the substrate. Although much progress has been made in our ability to repurpose aminoacyl-tRNA synthase to attach non-canonical amino acids to engineered tRNAs, [141,142] Harding et al. found a potential way to circumvent the use of aa-tRNAs as obligate substrates for CDPS-based catalysis: Using the *Bacillus thermoamylovorans* enzyme, BtCDPS they searched for minimal substrate requirement and found that activated amino esters can also function as substrates (although with poor catalytic rates) [143] and this finding was later verified with other CDPSs [124]. Their structure-based docking studies could potentially guide the way to engineering enzymes that are better catalysts of non-aa-tRNA substrates [143]. Further, Canu et al. showed via shortened tRNAs (so-called minitRNA) loaded with amino acids by using flexizyme (flexizyme-aminoacylated minitRNAs) that the acceptor's arms are the only parts of the tRNAs required for CDPS activity, setting the stage for better understanding and engineering minimal substrate requirements [144].

### 3.4 Mixing and matching: combining CDPSs and tailoring enzymes from different pathways

Another approach to creating new-to-nature cyclodipeptides is by co-expressing CDPSs with tailoring enzymes from other gene clusters. Some tailoring enzymes are very specific for their own dipeptide – such as the mycocyclosin-gene cluster encoded cytochrome P450 CYP121 that catalyzes an intramolecular C—C bond formation only within cYY and no other aromatic cyclic dipeptide substrate [79,145] – while others are more promiscuous and modify a set of substrates. Chevalier et al. showed that the combinatorial co-expression of bio-mined cyclodipeptide oxidases [CDOs] with CDPSs from a set of green clusters yields a variety of new chemistry [146]. In total the authors tested 144 combinations, stemming from 18 CDPSs in combination with 8 CDOs using direct LC-MS/MS-based analysis of the peptides in culture supernatants as the read-out, setting the first step towards synthetic gene cluster design and combinatorial engineering of new cyclodipeptides. Currently, there is limited knowledge on how certain chemistries affect function and creating and probing structure-function might help to achieve a more forward design of new cyclodipeptide encoded functionalities.

## 4 Strategies for the high-yield bioproduction of cyclodipeptides in bacteria and yeast

While the natural and engineered chemical diversity of cyclodipeptides holds promise for various applications, a bottleneck for cyclopeptide employment in agriculture or medicine is the limited access to sufficient amounts [147]. In the following, we discuss strategies to achieve high-yield production of cyclodipeptides via fermentation optimization and metabolic engineering using pulcherriminic acid (CDPS-produced) and thaxtomin A and its derivatives (NRPS-produced) as examples. While for thaxtomin also chemical synthesis routes are available, low production yield, environmentally damaging waste products, and racemic mixtures make bioproduction a more sustainable and economical route [147,148,64].

### 4.1 Optimization of cyclodipeptide production via fermentation optimization

Optimization of fermentation conditions is still one of the most critically explored paradigms before any large-scale metabolite synthesis, and it is an effective method for improving bioproduction [154]. As opposed to metabolic engineering it is a GMO-free way to increase the yield of the desired product. The optimization should include physical conditions such as pH, temperature, agitation speed, and appropriate medium conditions such as carbon source, nitrogen source, C/N ratio, ionic strength, and substrate concentration. Achieving high yield production of cyclodipeptides through fermentation condition optimization has been extensively studied, especially for pulcherriminic acid and thaxtomins [147,151,155].

#### 4.1.1 Optimizing fermentation conditions for pulcherriminic acid production

Pulcherrimin – the name for pulcherriminic acid when bound to iron **(**Fig. 5**D)** – can be detected by the naked eye because of its brown color. It can be quantified by purifying the insoluble pulcherrimin from the culture supernatant (solubilization via alkaline methanol [110]) followed by UV–Vis spectrometry via its characteristic absorption spectrum. This allows for production assessment across various conditions, although in a rather low throughput [153,156]. During the early discovery of the molecule, it was shown that the addition of ferric ammonium citrate raised the production of pulcherrimin in *Metschnikowia pulcherrima* [previously known as *Torulopsis pulcherrima*) and optimal physical conditions were shown to be a low pH (pH4) [110,118]. In terms of temperature condition, Roberts et al.*,* stated that the total production of pulcherrimin is probably not affected by temperature, but temperatures below 16 °C or 19 °C seem to favor cellular retention, while higher temperature favor pigment diffusion [152]. In contrast to Roberts et al., Horvarth et al. showed in solid media that higher temperature decrease the diameter of the red pigmented zone which means that higher temperature decreases pigment diffusion. However, in broth media at pH 4.7 and 5, the higher temperatures increased the production of pulcherrimin [155]. It seems that the effect of temperature in the production of pulcherrimin is still unclear and depends on the culture conditions, thus more investigation is needed.

In accordance, Horvarth et al. found that also for the yeast *Metschnikowia andauensis,* pulcherrimin production and localization (extra versus intracellular) was highly influenced by media and cultural conditions [155]. Glucose, galactose, disaccharides, and the presence of pectin or certain amino acids promoted the production. At the same time, there was no pigment produced with mannose, fructose, or sorbose as the carbon source. The presence of specific amino acids such as leucine, glutamic acid, arginine lysine, serine, threonine, alanine, and cyclo(L-leu-l-leu) in the medium strongly increases the pulcherrimin production [155].

In *Bacillus* spec., glucose was found to be the best carbon source and ammonium disulfate the best nitrogen source to promote pulcherrimin production. In both, bacteria (*Bacillus licheniformis*) and yeast (*Metschnikowia* spec), the addition of surfactant (Tween 80) increased extracellular amounts of pulcherrimin – likely due to increased cell membrane permeabilization – as well as citrate promoted overall production [151,153]. It is noteworthy that all studies discussed above were performed with different isolates of *Metschnikowia* spec. *or Bacillus* spec*.* and optimal conditions might vary across these isolates.

#### 4.1.2 Optimizing fermentation conditions for thaxtomin production

For the production of thaxtomins, fermentation conditions in the natural producer *Streptomyces scabies* have been investigated. The production of thaxtomins is mainly analyzed and quantified by HPLC followed by liquid chromatography-mass spectrometry (LC-MS) analysis [147,149]. However, the typical yellow color can serve as a simpler and more high-throughput-compatible preliminary screening readout [149,157]. As the production of the compound is related to the *Streptomyces scabies* pathogenicity towards host plants, plant extract containing media was shown an important factor to stimulate thaxtomin production, with the plant polymer suberin likely being one of the molecular inducers for bioproduction [51]. Specifically, oat bran broth (OBB) showed the highest production across various strains of *Streptomyces scabies* [36] and this medium has since been used in various studies to produce thaxtomins by pathogenic *Streptomyces* strains for further investigation [51,59,60,158]. It was further shown that cellobiose induces thaxtomin production when added to a variety of different growth media typically used for the growth of *Streptomyces* strains [147]. The molecular mechanism of cellobiose-induced thaxtomin production works via induction of CebR – the master-regulator of cellulose/cellobiose/cell-ologosaccharide utilization in multiple *Streptomyces* species – that also positively regulates the *txt* gene cluster repressor TxtR, which is encoded by the cluster-encoded gene *txtR* Fig. 3 [147,66,159]. TxtR belongs to the ARAC/XylS family. Of transcriptional regulator and it functions as an activator of *txtA, txtB, txtC, txtD* expression [66]

### 4.2 Optimization of cyclodipeptide production via metabolic engineering

Metabolic engineering has become an essential approach in developing high-performance microbial cell factories for the production of various chemicals. Many strategies are now available that combine systems biology, synthetic biology, evolutionary engineering, and machine learning and that allow cheaper, and quicker ways to engineer pathways [160,161,162].

Metabolic engineering is highly dependent on molecular knowledge of the biosynthetic pathway of the compound as well as on the availability of genetic tools for engineering the producer strain or the possibility to functionally express the gene cluster in a heterologous host. Since several important cyclodipeptide metabolic pathways have been elucidated recently, efforts to engineer the pathways have become possible. Although we focus on pulcherriminic acid [152] and thaxtomin A [149], other pathways such as the one for bacitracin production have been engineered as well [163]. Noteworthy, since the biosynthesis of pulcherriminic acid and thaxtomin A rely on two unrelated biosynthetic pathways – CDPS and NRPS-based respectively – the engineering strategies are different. For example, in the CDPS pathway, overexpression of specific aminoacyl-tRNA synthase, which are the substrates for the CDPS scaffolds is a promising strategy. Still, several common strategies were successfully applied such as heterologous pathway expression, repressor deletion, and operon and gene clusters overexpression via promoter replacements [149,152,163].

#### 4.2.1 Metabolic engineering of thaxtomin production

As described above, the NRPS-based gene cluster for thaxtomin A (*txt* gene cluster) is well studied. The fact that it is encoded in plant-pathogenic *Streptomyces* species (*S. scabis, S. acidiscabis, S. turgidiscabis*), as well as the low production levels in these strains, hampers its bioproduction potential. In a first engineering step, Jiang et al. [147] functionally ported the 18-kb gene cluster (cloned on a integrative bacterial artificial chromosome, BAC) into the non-pathogenic species *Streptomyces albus*. This strain achieved around ten times higher production yields (91.2 ± 6.8 mg/liter) when compared to the native host *S. scabiei* 87.22 (9.1 ± 0.4 mg/liter). After media optimization, the strain produced up to 222 mg/ml thaxtomin analogs **(**Table 1). Again, cellobiose was used as the inducer molecule to activate the cluster expression via CebR (see above). The authors further showed that various thaxtomin biosynthetic intermediates could be produced by engineering the gene cluster via gene deletions. Thaxtomin intermediates can function as precursors for the semisynthesis of advanced thaxtomin analogs [148,164,165]. For example, the intermediate thaxtomin D could be produced (75.9 ± 1.1 mg/liter) by deletion of *txtC*, the P450 monooxygnease catalyzing the last hydroxylation step in thaxtomin A production **(**Fig. 3). Further, nitrotryptophans could be produced (56.2 ± 3.5 mg/liter) by deleting *txtABC* and *H* thus just allowing the first step in thaxtomin A production, namely the 4-nitration of l-trp by the Cytochrome P450 monooxygenase TxtE supported by the nitric oxide synthase TxtD **(**Fig. 3**)**.

**Table 1:** Overview of strategies to optimize the production of cyclodipeptides.

Compounds	Host organisms	Strategies	Yields	Ref.
Thaxtomins	*Streptomyces albus* J1074	heterologous expression and medium optimization	222 mg/L	[147]

	*Streptomyces albidoflavus* J1074	heterologous expression, repressor deletion, activator overexpression, optimization of fermentation media and scale-up	1973 mg/L	[149]

*Streptomyces coelicolor* M1154	Heterologous expression, multiplexed promoter engineering	504.6 mg/L	[150]

Pulcherrimin	*Bacillus licheniformis* DW2	Medium optimization (Glucose, (NH4)2SO4, 0.1% Tween20)	331.17 mg/L	[151]
	*Bacillus licheniformis* DW2	Increasing precursor supply and overexpression of leucyl-tRNA synthase gene, promotor replacement	556.1 mg/L	[152]
	*Metschnikowia pulcherrima*	Medium optimization (Glucose, 0.1% (v/v) Tween20)	230–240 mg/L	[153]
	*Metschnikowia sinensis*	Glucose, 0.1% (v/v) Tween20	230–240 mg/L	[153]

Building on this first version of a successfully engineered thaxtomin-producing *S. albus J1074* strain*,* Li et al. performed a combinatorial engineering approach to further enhance production [149]. The authors first screened various *Streptomyces* hosts for heterologous production yield but confirmed that the *S. albus J1074* was the best producer. Using this strain, the authors then identified and deleted a homolog of the CebR master regulator and showed that this enabled thaxtomin A production (164 mg/liter) without the addition of the costly cellobiose. A similar result could be achieved when overexpressing the *txt* regulator TxtR by using a series of strong *S. albus* endogenous promoters that were identified in a promoter library approach (confirming the *rpoD* promoter to be a potentially universal strong promoter in *Streptomycis* species). qPCR analysis showed that the cluster was indeed highly overexpressed in the “unregulated” engineered strain. The combination of the CebR deletion and TxtR overexpression however did not yield additive effects, showing that maximal *txt* cluster expression could be achieved with either approach alone. Major yield improvements however could be achieved via media optimization and scale-up in a 5 L stirred-tank bioreactor to a final concentration of 1973 mg/liter **(**Table 1).

In a very recent, similar approach Zhao et al. [150] heterologously expressed the *txt* gene cluster from *S. acidiscabis* ATCC 49,003 in three well-characterized *Streptomyces* hosts (*S. venezuelae* ISP5230, *S. albus* J1074, *S. coelicolor* M1154). They enhanced production by refactoring the gene cluster using strong constitutive promoters and by constructing a pathway library using promoters of different strengths to drive expression of the three operons *txtED, txtABH*, and *txtC* to balance expression levels. Their efforts yielded a production of 504.6 mg thaxtomins/L **(**Table 1).

Another possible engineering strategy to potentially further increase the thaxtomin production is by increasing the supply of the amino acid precursors l-arginine, l-tryptophan, and l-phenylalanine. This strategy has been applied to increase the production of other cyclic peptides, such as bacitracin [cyclic dodecapeptide and pulcherriminic acid (cyclic dipeptide) [152,163]. Indeed, many studies have already shown the metabolic engineering of various bacteria towards increased amino acid production [166], [167], [168], [169], [170] These metabolic engineering efforts however aimed for increasing the production of extracellular amino acids. However, in the case of cyclodipeptide production, the amino acids used for cyclodipeptide synthesis should be intracellular amino acids. Thus, the increasing flux for amino acid uptake and decreasing flux for amino acid export will be beneficial to increasing the production of cyclodipeptides [166].

#### 4.2.2 Metabolic engineering of pulcherriminic acid production

**Fig. 6:**
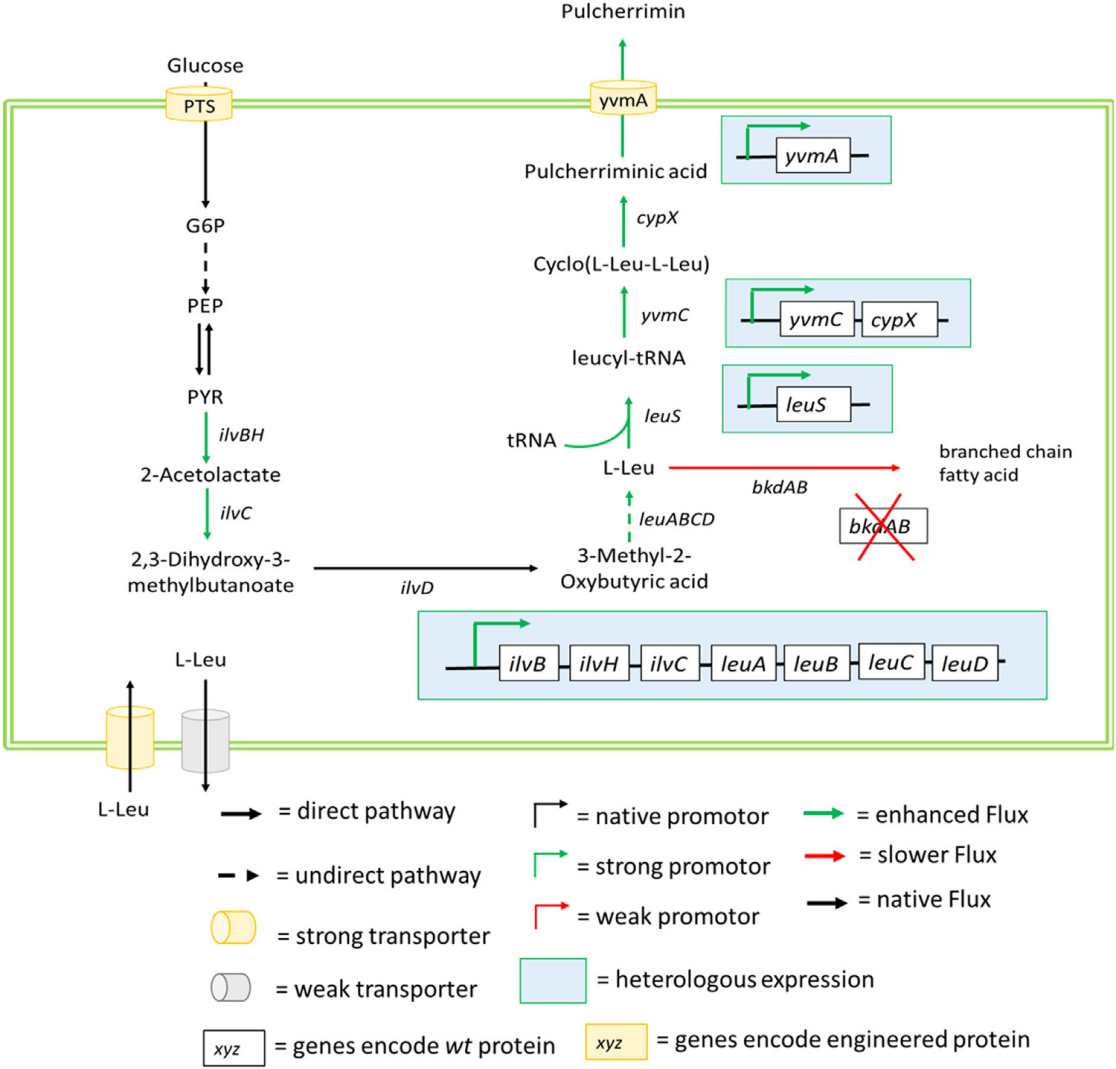
**Strategies for increasing pulcherriminic acid production in *Bacillus* spec***.* Green arrows indicate overexpression. Overexpression of the ilvBHC operon and the leuACD operon as well as deletion of a leucine-drainage reaction – catalyzed by the alpha-keto acid dehydrogenase bkdAB to increase intracellular l-leucine levels. Overexpression of the leucyl-tRNA synthase. (LeuS) to increase intracellular levels of leucyl-tRNA. Overexpression of the CDPS (encoded by *ymcC*) and the tailoring P450 oxygenase (encoded by *cypX*) and the exporter (encoded by *yvmA*) to increase pulcherriminic acid production and export [152,172,173]. Steps to control the export of overproduced leucine to enhance intracellular levels have not yet been undertaken.

Pulcherriminic acid is a prospective biocontrol agent synthesized by bacteria (*Bacillus* spec) and yeast (*Meschnikovia spec*). Biosynthesis and some engineering efforts for enhancing pulcherriminic acid production in *Bacillus* have been reviewed recently and the main strategies are summarized in Fig. 6 [29]. Here we summarize these efforts and point to new opportunities for engineering pulcherriminic acid production in yeast, for which a biosynthetic gene cluster was recently described [105]. Wang et al. started their engineering endeavor in *B. licheniformes* by showing via external leucine supplementation that leucine was indeed a limiting factor in pulcherriminic acid biosynthesis. [152] Consequently, they successfully enhanced intracellular leucine levels by placing the leucine production-relevant genes and operons *ilvD, ilvBHC,* and *leuABCD* under control of the previously shown strong promoter P_bacA_, derived from the bacitracin biosynthetic gene cluster. The authors performed their engineering via CRISPR/Cas9. Like this, the authors achieve a leucine overproduction of 173%, enhancing the pulcherriminic acid production from 127.5 mg/liter to 149.9 mg/liter. Interestingly they show via an engineered LeuA [the R529H/G532D double mutant that is less sensitive to feedback inhibition) that these levels are not yet high enough for feedback control to play a major role. The further deletion of a leucine-drainage reaction – catalyzed by the alpha-keto acid dehydrogenase bkdAB – enhanced pulcherriminic acid production to 189.9 mg/liter. Steps to control the export of overproduced leucine to enhance the intracellular levels have not yet been undertaken.

As mentioned earlier, the actual substrate for cyclic dipeptide biosynthesis via the CDPS is not the two amino acids themselves [in the case of pulcherriminic acid two leucine molecules) but rather their aa-tRNA (two leucyl-tRNAs in the case of pulcherriminic acid). As such the authors show that overproduction of LeuS, the leucyl-tRNA synthase, increased pulcherriminic acid production further to 367.7 mg/liter. Pulcherriminic acid secretion could then be enhanced to 556.1 mg/ml by overexpression of the transporter protein yvmA **(**Table 1).

Concerning engineering the regulation of pulcherriminic acid production, in *B. subtilis*, the YvmB MarR-like regulator is considered the main regulator for the production of PA by binding to the promoter region of *yvmC* and thus inhibiting *yvmC-cypX* cluster expression [171]. Another identified regulator cluster is AbrB-YvnA-YvmB [171]. As this dual regulation is rather complex and can affect global gene expression, the authors argue that their promoter replacement and overexpression approach of the yvmC-yvmX gene cluster is a more targeted approach to counteract repression of the gene cluster [152].

Until today, metabolic engineering for pulcherriminic acid production has only been conducted in *Bacillus* species. Efforts for engineering yeast have been restricted by the limited knowledge of the biosynthetic pathways leading to pulcherriminic acid production in yeast. Recently Krause et al. successfully identified the gene cluster for pulcherriminic acid biosynthesis in *K. lactis* and other yeast [105]. However, as the enzyme Pul1 – which seems to be responsible for cyclo(L-Leu-l-Leu) generation – is not phylogenetically related to any CDPS, it is still unclear what mechanism of action the enzyme follows and whether it uses Leucyl-tRNAs as precursors. Thus, more molecular details are required for effective metabolic engineering. Without any genetic engineering, the current highest yield of pulcherriminic acid production was achieved in an isolate of *M. pulcherrima* (240 mg/L). [153] By overexpressing the *PUL1* and *PUL2* genes via promotor replacement or increasing the supply of l-leu via metabolic engineering, may increase the pulcherriminic acid production in the natural producers or heterologous hosts. In terms of regulation, recently, Snf2 has been found to regulate *PUL1* and *PUL2* expression in *M. pulcherrima*. Snf2 is a non-essential ATPase in *S. cerevisiae* that alters nucleosome confirmation as part of the SWI/SNF chromatin remodeling complex, hence regulating the transcription of many genes [174]. Snf2 deficiency may affect chromatin remodeling at the *PUL1, PUL2*, and *PUL3* loci, preventing the adequate transcription of the genes [175]. As such, the expression of both genes from strong constitutive promoters that are not regulated by Snf2 should make expression stronger and growth-state independent.

## 5 Synthetic biology and cyclodipeptides

In this section, we will discuss synthetic biology tools that could aid the metabolic engineering of cyclodipeptide production and enhance their chemical diversification. Synthetic biology, protein engineering, and metabolic engineering rely on one another to thrive and accomplish their aims [176], [177], [178], [179]. The goal of synthetic biology is to create libraries of genetic components (promoters, coding sequences, terminators, transcription factors and their binding sequences, and more), to assemble devices, genetic circuits, and even (re-)designed organisms, and to retrieve quantitative data to develop models that can predict the behavior of biological systems [180]. Metabolic engineering aims to maximize existing and synthetic cellular processes to synthesize the desired product from an ideally inexpensive and simple substrate [177]. The goal of protein engineering within the context of metabolic engineering mostly focuses on enzymes and biosensors [181,182]. It reaches from changing the substrate specificity of an enzyme or receptor, enhancing catalytic rates, and dynamic ranges, eliminating inhibition; changing physicochemical properties (e.g. solubility, stability), but also designing entire new enzymes or sensors with new-to-nature functions.

The overall workflow in synthetic biology and metabolic- and protein engineering relies on the so-called **design-build-test-learn** (DBTL) cycle: **1)** A new biological part or system with the desired output is first **designed** *in silico* based on previous knowledge and/or mathematical and computational modeling. Often many possible designs are created that are channeled into the DBTL cycle to enhance the chance that one of the designs functions as predicted. **2)** The designs are then **built** “in DNA” by assembling required genes, circuits, and pathways on plasmids or by engineering organisms on the genomic DNA level. **3 and 4)** The designs are then tested with appropriate assays and the results and best designs are fed back into the DBTL cycle for further improvement. The goal of synthetic biology has been to provide tools and resources to run the cycle as efficiently as possible and to reduce the rounds required to achieve an engineering goal.

Several excellent reviews have focused on available synthetic biology and metabolic engineering strategies and tools to enhance the chemical diversity and the biosynthetic yields of natural and unnatural products [160,161,[183], [184], [185], [186]]. Here we just highlight those advancements that we think will be most relevant for the field of cyclodipeptides and highlight where these techniques have already been applied for cyclodipeptide engineering. We organized them according to their relevance within the DBTL cycle.

### 5.1 Learn and design: metabolic engineering and protein engineering enhanced by machine learning

As shown above, for cyclodipeptide production, both, fermentation conditions and metabolic fluxes play a role when trying to enhance the production levels in natural and heterologous hosts: the search space for finding an optimal solution is thus given by multiple parameters and therefore huge. Huge search spaces are a general challenge for any metabolic engineering or protein engineering project as they require often-not-available high-throughput methods to search them.

Recently, machine learning tools have been developed that guide the experimenter through this large search space [162,187,188]. These tools can use a relatively small initial data set to learn from and make suggestions on better designs toward a user-defined output.

These tools facilitate the engineering of biology more time-and resources effectively by reducing the required rounds in the design-build-test-learn cycle to reach (near)optimal solutions for an engineering challenge. [189] In the context of cyclodipeptides, these tools could help to improve precursor and thus cyclodipeptide production via metabolic and fermentation engineering.

### 5.2 Build: well-characterized modular cloning toolkits and genome engineering tools for (un)conventional bacteria and yeast

To tune the expression levels of the enzymes within a (re)designed metabolic pathway – an essential prerequisite for generating data that can be used by the machine learning algorithms mentioned above – modular cloning toolkits have proven very useful. These toolkits are collections of well-characterized promoter and terminator parts as well as expression vectors or CRISPR-based engineering tools that can be used to assemble pathways in a modular fashion based on type IIS restriction enzyme cloning. Those kits have been developed and characterized for various model organisms and start to be developed for less conventional bacteria and yeast [190], [191], [192], [193], [194]. In the context of cyclodipeptide engineering, Li et al. started to create a well-characterized promoter library for *S. albus* that proved to be useful to overexpress the thaxtomin gene cluster regulator TxtR [149]. Repurposing the existing toolkits or creating additional ones could aid the metabolic engineering of high-yield producers, especially when involving machine learning.

### 5.3 Build: cloning and (de-novo) assembly of gene clusters for expression in heterologous hosts

When building cell-factories for the production of cyclic dipeptides one can either engineer the natural producer strain or clone the gene cluster into a heterologous host. As outlined above, most of the fermentation optimization for pulcherrimic acid production has been conducted in the natural production hosts (e.g. *Meschnikowia, Kluveromycis*, and *Bacillus*), while thaxtomin production has been mostly optimized in a non-pathogenic heterologous production host. The advantage of using a heterologous production hosts such as the yeast *S. cerevisiae* or the bacterium *E. coli* is that well-established tools for boosting production by going through the DBTL cycle – such as metabolic models and genetic engineering tools – are available and that they are non-pathogenic. However, heterologous production requires that the full gene clusters need to be identified and cloned. Several recent reviews highlight advances and tools for the reconstruction and heterologous expression of natural product biosynthetic gene clusters. [195,196] Further, it can become useful to synthesize the gene clusters de-novo rather than copying individual genes from the genome of an organism via PCR. This is explicitly relevant when individual genes need to be codon optimized for use in a heterologous host or the cluster structure needs to be refactored for optimized expression (as shown for the heterologous *txt* gene cluster expression [150]). Various methods for the de-novo assembly of genes, pathways and entire genomes are available: For example, yeast assembly – which is based on homologues recombination in the yeast *S. cerevisiae* - has been optimized to assembly pathways and entire genome *“*in vivo*”*. [197] Isothermal assembly (also known as Gibson assembly) is a powerful method to assemble linear fragments into pathways and genomes in vitro*.* [198] Starting points for both, yeast assembly and isothermal assembly are linear double stranded fragments of several hundred base pairs which are commercially available, however, methods for affordable in-house assembly of these starting fragments from chemically synthesized oligonucleotides have recently been developed and could also be used to clone cyclodipeptide gene clusters. [199], [200], [201], [202], [203], [204]

### 5.4 Build and test: cell-free synthetic biology to rapidly create a toolbox of well-characterized enzyme reactions and for combinatorial bioproduction

Cell-free synthetic biology has become a mature technique for rapid characterization of biological parts [205,206] – including enzymes [207,208,209] – but also the actual high-yield bioproduction of small molecules and proteins [210,211]. Cell-free approaches have several advantages compared to fermentation in living cells, including the ability to control which components are part of a system, to minimize side reactions, and achieve higher yield and productivity, especially of compounds that are toxic to living cells [212,213]. Cell-free (or in vitro) approaches have already been applied to the field of cyclodipeptide biosynthesis: Jiang et al. reported the in vitro biocombinatorial synthesis of thaxtomin analogs using purified enzymes from the txt gene cluster that had been expressed in *E. coli* [64]. Noteworthy this approach works with purified enzymes rather than using the enzymes directly in cell-free extract, but it is imaginable that the approach is compatible with the latter. Furthermore, Goering et al. express GrsA and GrsB1 – encompassing the first two of five modules of gramicidin S biosynthesis – in a cell-free system and report higher peptide production yield than reported for cell-based expression [214].

Currently, the rate at which new cyclodipeptide biosynthetic pathways are identified by genome mining outpaces the current capacity to experimentally characterize the encoded enzymes and their products. Substantial efforts have already been made to characterize CDPS and tailoring enzymes in vivo. [93,86,146] Cell-free characterization is medium-to-high throughput compatible and could become an alternative way to gain experimental data on substrate specificity product formation of natural and engineered enzymes. Further, existing approaches for combinatorial biosynthesis of cyclodipeptide using enzymes from different pathways in vivo [146] could be facilitated in cell-free systems. Here a single cell-free system needs to be prepared that can then be spiked with linear DNA encoding for combinations of CDPS and tailoring reactions, circumventing laborious transformation and culturing. Best performing reactions can then be scaled in vivo or in vitro.

### 5.5 Test: high-throughput analysis of created designs via biosensors

Metabolic engineering and protein engineering rely on the ability to evaluate a large number of designs in parallel to read out the amount of produced product; a specific cyclodipeptide or intermediate substrate. To address this challenge many synthetic biology efforts have focused on building biosensors based on engineered transcription factors, [215] riboswitches and toehold switches, [216,217] or transmembrane receptors [218,219] and using them in different modalities to create medium to ultra-high-throughput assays for evaluation [220,221].

Chevalier et al. developed a medium-throughput method based on LC-MS/MS- to detect combinatorically produced cyclodipeptide directly in the culture supernatant of producing *E. coli* strains [146]. In addition available sensors that measure the intracellular concentration of specific amino acids, [222,223,224] or peptide- or small molecule sensing GPCRs [225,226,227,228] might be engineerable to detect secreted cyclodipeptides might be useful for the optimization of precursor and/or final product synthesis.

## 6 Discussion

Here we summarized recent research on the biosynthesis of cyclodipeptides, as well as strategies for expanding the peptide's chemical diversity, and approaches to increase their production in cell factories.

Much progress has been made over the last decades in understanding how NRPS-based cyclodipeptide assembly lines can be engineered and supplemented with non-canonical substrates to yield non-natural peptide chemistries. In addition, the field of CDPS-encoded cyclodipeptides - while much younger than the NRPS-base field - is gaining momentum. The first well-characterized enzyme toolboxes for combinatorial biosynthesis have been made available, first directed evolution experiments show the engineerability of the enzymes, as well as non-natural substrates for chemical diversification, have been identified. The small size of the CDPS enzymes themselves (∼30 kDa in comparison to >100 kDa) but also the small size of the biosynthetic gene clusters makes them promising tools for biosynthesis.

Genome mining of cyclodipeptide gene clusters points to the expected diversity in encoded new tailoring reactions. To experimentally characterize and exploit this catalytic diversity, medium-to-high throughput assays such as developed in the field of cell-free synthetic biology could yield great setups to characterize enzymes and prototype combinatorial new-to-nature biosynthetic pathways [207], [208], [209]

Another challenge for the translation of cyclodipeptides into marketable agricultural, food, or medical product is their bioproduction in large amounts. Several studies show that metabolic engineering and fermentation optimization hold promise to create suitable cell factories. Advanced machine learning tools for protein engineering, metabolic engineering, and fermentation optimization, [188,162] as well as the development of high-throughput screening systems based on biosensors could accelerate these ongoing efforts [215,220]. Also, production processed in cell-free systems could be further explored. [214]

There is no doubt that cyclodipeptides are interesting molecules in terms of structural diversity and bioactivities. While methods are maturing to increase the chemical space – and thus the functional space – of cyclopeptides and enhance their bioproduction, the question remains which molecules likely have useful activities and should be focused on for biosynthesis. Here recent advances in using deep learning to facilitate the prediction of drug-target interactions [229] and antibiotic discovery [230] could be useful tools for prioritizing which chemical space to explore.

## Declaration of Competing Interest

The authors have no financial and personal relationships with other people or organizations that could inappropriately influence (bias) their work.
